# IL-33 signaling is essential to attenuate viral-induced encephalitis development by downregulating iNOS expression in the central nervous system

**DOI:** 10.1186/s12974-016-0628-1

**Published:** 2016-06-22

**Authors:** Rafael F. O. Franca, Renata S. Costa, Jaqueline R. Silva, Raphael S. Peres, Leila R. Mendonça, David F. Colón, José Carlos Alves-Filho, Fernando Q. Cunha

**Affiliations:** Department of Virology and Experimental Therapy LAVITE, Oswaldo Cruz Foundation – FIOCRUZ, Institute Aggeu Magalhães IAM, Av. Professor Moraes Rego, s/n, Recife, PE 50740-465 Brazil; Program of Basic and Applied Immunology, University of São Paulo, Ribeirao Preto, SP 14049-900 Brazil; Department of Pharmacology, Ribeirão Preto School of Medicine, University of São Paulo, Ribeirão Preto, SP 14049-900 Brazil

**Keywords:** Interleukin 33, ST2 receptor, Experimental viral encephalitis, Nitric oxide, Flavivirus

## Abstract

**Background:**

Viral encephalitis is a common cause of lethal infections in humans, and several different viruses are documented to be responsible. Rocio virus is a flavivirus that causes a severe lethal encephalitis syndrome in humans and also mice, providing an interesting model to study the CNS compartmentalized immune response. Interleukin 33 (IL-33), a member of the IL-1 family, is an immunomodulatory cytokine that is highly expressed in the CNS. However, the role of IL-33 on viral encephalitis remains unclear. Therefore, we aimed to explore how the IL-33/ST2 axis regulates the local immune response during Rocio virus infection.

**Methods:**

Wild-type (WT), ST2 (ST2^−/−^), and nitric oxide synthase-deficient mice (iNOS^−/−^) and Stat6 (Stat6^−/−^)-deficient mice were infected with different concentrations of the Rocio virus by intraperitoneal route, the cytokine mRNA level in CNS was analyzed by qPCR, and cellular immunophenotyping was performed on infected mice by the flow cytometry of isolated CNS mononuclear cells.

**Results:**

We have shown that the mRNA expression of IL-33 and ST2 receptors is increased in the CNS of Rocio virus-infected WT mice and that ST2^−/−^ mice showed increased susceptibility to infection. ST2 deficiency was correlated with increased tissue pathology, cellular infiltration, and tumor necrosis factor alpha (TNF-α) and interferon-gamma (IFN-γ) mRNA levels and higher viral load in the CNS, compared with wild-type mice. The increased Th1 cytokine levels released in the CNS acted on infiltrating macrophages, as evidenced by flow cytometry characterization of cellular infiltrates, inducing the expression of iNOS, contributing to brain injury. Moreover, iNOS^−/−^ mice were more resistant to Rocio virus encephalitis, presenting a lower clinical score and reduced mortality rate, despite the increased tissue pathology.

**Conclusions:**

We provide evidences of a specific role for IL-33 receptor signaling in nitric oxide induction through local IFN-γ modulation, suggesting that nitric oxide overproduction might have an important role in the progression of experimental viral encephalitis.

**Electronic supplementary material:**

The online version of this article (doi:10.1186/s12974-016-0628-1) contains supplementary material, which is available to authorized users.

## Background

The Rocio virus (ROCV), member of the Flaviviridae family with some well-known viruses, such as West Nile, dengue, yellow fever, and more recently Zika, is a single-stranded RNA virus that infects humans and other vertebrates. Infection with ROCV leads to a wide range of symptoms including, but not restrict to, the following: fever, headache, anorexia, nausea, vomiting, myalgia, and malaise. The pathology of this viral infection in humans ranges from asymptomatic to acute encephalitis [1]. Previous studies from our group demonstrated that ROCV infects and induces in BALB/c mice damage of the central nervous system (CNS), characterized by neuronal degeneration and apoptosis with detectable levels of inflammatory cytokines and massive cellular infiltration [2]. Moreover, cellular infiltration in CNS is highly dependent of the CCR-5/MIP-1α axis, since CCR5 and macrophage inflammatory protein 1 alpha (MIP-1α) knockout mice presented reduced inflammation with increased survival rates after ROCV infection [3]. CNS cellular infiltration induced by ROCV infection is characterized by F4/80^+^ cells, detected early as 4 days post-infection. At later points after infection, the CNS became infiltrated with NK, B cells, and especially T lymphocytes. Locally, these cells produce high amounts of interferon-gamma (IFN-γ) and tumor necrosis factor alpha (TNF-α), among other cytokines, which contribute to the degeneration and death of neurons with extensive tissue damage [2].

Interleukin 33 (IL-33), a member of the IL-1 cytokine family, is constitutively expressed by tissue barrier cells such as the epithelial and endothelial cells of different body compartments. In addition, some innate immune cells such as macrophages and dendritic cells also express IL-33 [4–6]. IL-33 is recognized as the functional ST2 receptor ligand, a heterodimer receptor complex consisting of ST2 and the IL-1 receptor accessory protein (IL-1RAcP) [4]. ST2 cell surface expression is observed on T helper 2 (Th2) cells but not on T helper (Th1) cells; thus, it functions as an important effector molecule of T helper type 2 responses [7]. ST2 activation signals through the myeloid differentiation primary response gene 88 (MyD88) and nuclear factor kappa b (NF-kB) pathway, inducing the production of several cytokines and chemokines. Moreover, ST2 activation causes cell differentiation, polarization, and activation, depending on the target cell [5].

On viral and parasitic experimental infections, IL-33 plays a crucial role in the local immune response to several different pathogens [8–11]. In fact, it has been reported that in mice, IL-33 is necessary for effective cluster of differentiation 8 (CD8^+^) T cell responses to replicate lymphocytic choriomeningitis virus (LCMV), an RNA virus, and against murine γ-herpesvirus 68 (MHV-68), a DNA virus. Interestingly, recombinant IL-33 administration improved vaccine-induced CD8^+^ T cell responses to vaccinia virus-based vectors and virus-like particles (VLPs) [12]. Following infection with coxsackievirus B (CVB), mice deficient for T1/ST2 (IL-33R signaling) significantly developed more severe pancreatitis with greater weight loss and higher viral load compared with wild type [8]. However, the role of IL-33 in the ROCV-induced host CNS-damage was not addressed yet. In human, rhinovirus infection induced asthma exacerbation dependent of IL-33 and the production of type 2 cytokines IL-4, IL-5, and IL-13. The authors state that IL-33 induction directly correlates with viral load and IL-5 and IL-13 levels, proposing IL-33 inhibition as a novel therapeutic approach for virus-induced asthma exacerbations [13]. However, depending on the organ involved and the Th1/Th2 immune balance necessary to control the infection, IL-33 may also present opposing roles.

Herein, we are showing that the ST2 receptor is expressed in the CNS during experimental murine ROCV-induced encephalitis. The ST2 deficiency, by target disruption in BALB/c mice (T1/ST2^−/−^), led to impaired control of viral burden in the CNS, which was associated with an early cellular CNS infiltration and a Th1-polarized immune response with consequent increased expression of the cytokines TNF-α and IFN-γ, which in turn upregulates tissue inducible nitric oxide synthase (iNOS) expression. In the brain, the generation of reactive nitrogen species through iNOS induction could act by increasing the oxidative stress responsible for tissue injury, worsening the disease. In conclusion, our data suggest that IL-33 might play a crucial role in the pathogenesis of virus-induced encephalitis by controlling the local Th1/Th2 balance.

## Results

### BALB/c mice are susceptible to ROCV infection

**Fig. 1:**
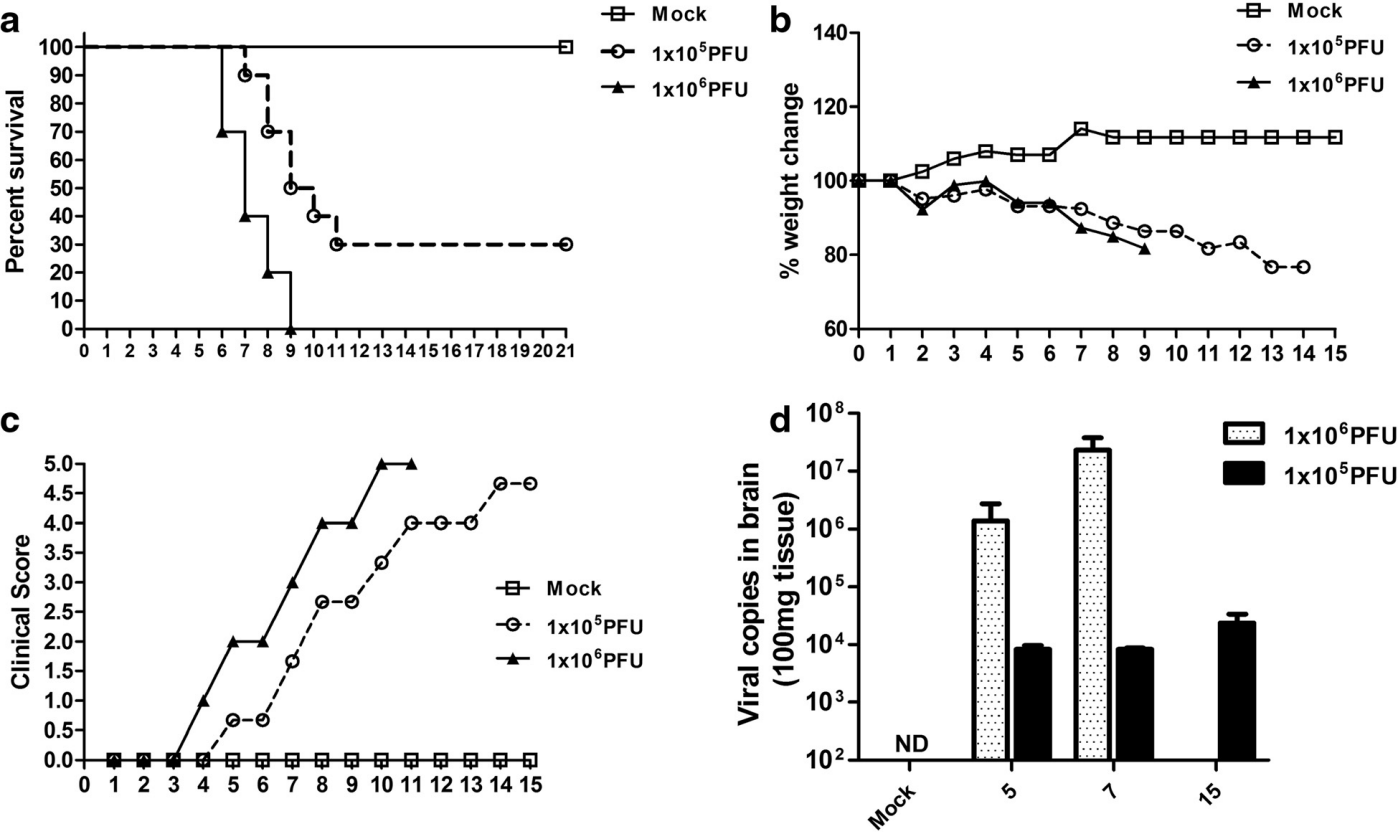
BALB/c mice are susceptible to ROCV infection. **a** Groups of ten female 6-week-old BALB/c (WT) mice were infected intraperitoneally (i.p.) with either 1.10^5^ or 1.10^6^ PFU of ROCV and monitored daily for survival up to 21 days post-infection. **b** Infected mice were monitored daily for weight loss after infection. **c** Clinical score of ROCV-infected mice, evaluated as described in the “Methods” section. **d** The viral load on the brains of infected mice was assessed by real-time qPCR on days 5, 7, and 15 post-infection. Animals were infected with 1.10^5^ or 1.10^6^ PFU of ROCV as indicated. Results are representative of the mean of ten animals per group. *ND* not determined

First, we assessed the susceptibility of BALB/c by infecting these animals with different concentrations of the Rocio virus (ROCV) by intraperitoneal (i.p.) route. By 9 days after i.p. inoculation, 1.10^6^ plaque-forming unit (PFU) ROCV-infected mice experienced 100 % mortality; in contrast, 1.10^5^ PFU ROCV-infected mice showed a reduced and delayed mortality. The peak of mortality was 70 % which was reached at 11 days post-infection (Fig. 1a). There are no differences on weight change on mice infected with 1.10^6^ or 1.10^5^ PFU (Fig. 1b); however, higher titer-infected mice displayed an earlier development of encephalitis signals, as determined by clinical score (Fig. 1c). We also recovered increased tissue virus concentrations on higher titer-infected mice, since this group presented a higher viral load on the brain, when compared to low titer infection (Fig. 1d).

### Infected BALB/c mice display high cell infiltration and cytokine production in the CNS

**Fig. 2:**
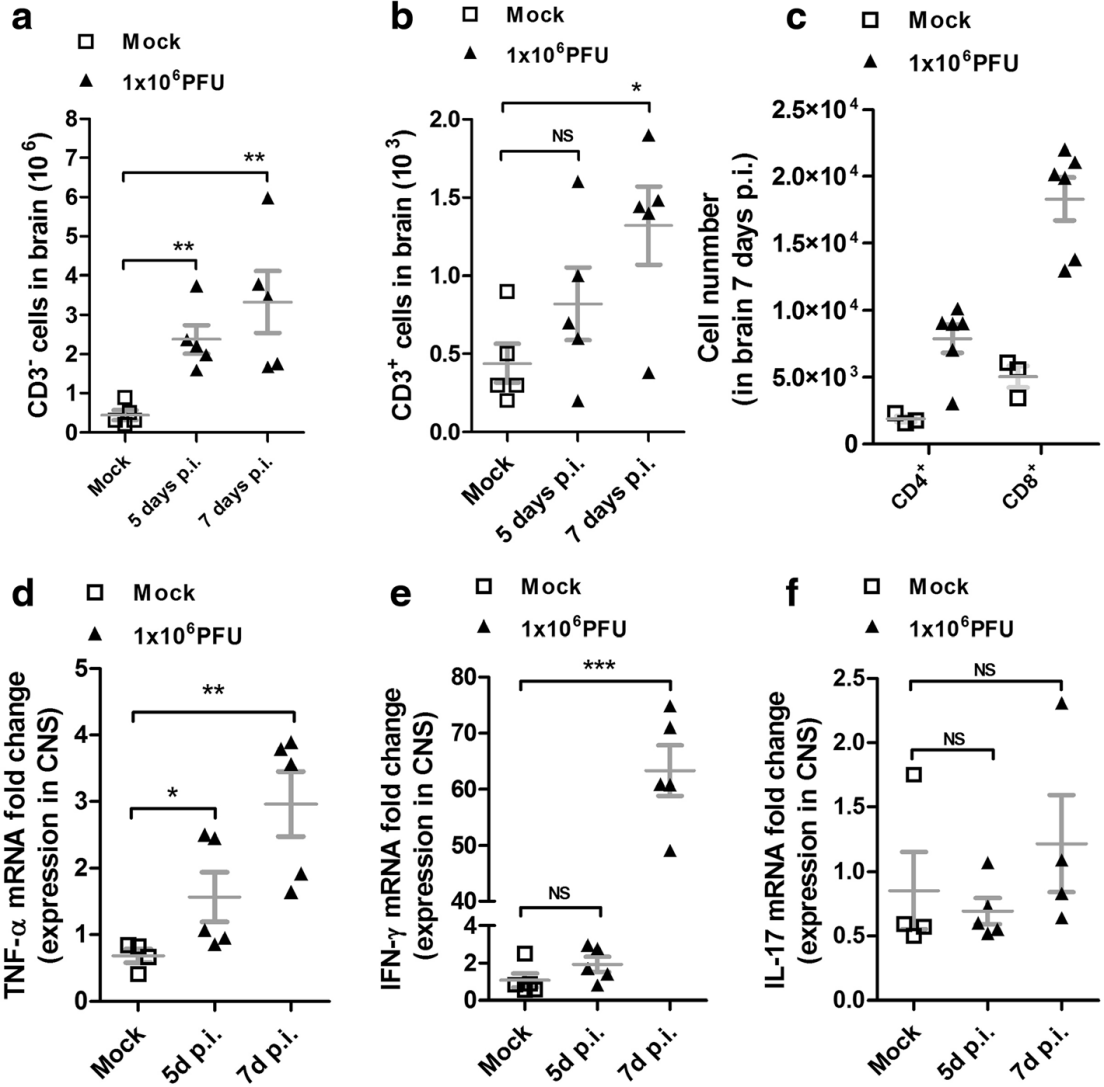
Inflammatory profile in the brain of ROCV-infected BALB/c mice. **a** Number of CD3^−^ cells in the brain after 5 or 7 days of infection with ROCV 1.10^6^ PFU analyzed by FACS. **b** Number of CD3^+^ cells after ROCV infection as indicated. **c** Number of CD4^+^- and CD8^+^-infiltrating cells in the brain after 7 days post-infection (disease peak) with ROCV 1.10^6^ PFU, gated on CD3^+^ cells. **d** Expression levels of TNF-α mRNA in the brain of mice infected with ROCV 1.10^6^ PFU 5 and 7 days post-infection, analyzed by qPCR. **e** qPCR of IFN-γ mRNA expression in the brains of ROCV-infected mice. **f** IL-17A mRNA expression. *p < 0.05, **p < 0.001 or ***p < 0.0001. *NS* non-significant. Data are representative as the mean of three independent experiments with at least four animals per group. Statistical analysis was performed by Student *t* test using the software GraphPad Prism

In two previous studies, we characterized partially the immune response to ROCV infection [2, 3]. Here, to better understand the immunopathologic process, we infected BALB/c mice with 1.10^6^ PFU ROCV, sacrificed these animals at 5 and 7 days post-infection (p.i.) (when encephalitis symptoms are evident) and analyzed the profile of cell infiltration in the CNS by fluorescent-activated cell sorting (FACS). We observed an increased cellular infiltration on CNS, characterized mainly by CD3^−^ cells at 5 and 7 days p.i. (Fig. 2a). In the cellular infiltrates, it was also demonstrated the presence of CD3^+^ cells (Fig. 2b), constituted in its majority by CD3^+^CD8^+^ cells and fewer CD3^+^CD4^+^ cells, and these infiltrates were significantly higher at 7 days p.i., coinciding with disease peak and clinical symptom appearance (Fig. 2c). Next, we analyzed the kinetics of the local immune response by quantifying the messenger ribonucleic acid (mRNA) expression level of T helper subtype signature cytokines IFN-γ and IL-17A and the pro-inflammatory cytokine TNF-α, in the CNS of ROCV-infected mice. The TNF-α mRNA level was significantly increased at 5 days p.i., reaching an even higher level by 7 days p.i. (Fig. 2d). At 7 days p.i., we observed a massive expression of IFN-γ mRNA (Fig. 2e), coinciding with the appearance of CD3^+^ on the CNS tissue (Fig. 2b, c) and signs of disease. As expected, the IL-17A cytokine (Fig. 2f) was not significantly altered on ROCV-infected animals, when compared to mock-infected animals.

### IL-33 is expressed by CNS-infiltrating F4/80^+^ cells

**Fig. 3:**
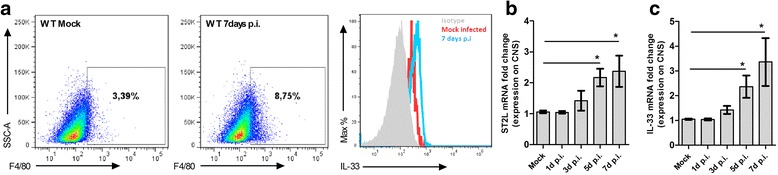
IL-33 expression in CNS-infiltrating cells. **a** Frequency of F4/80^+^ cells in the CNS of false infected WT mouse (WT mock), analyzed by FACS. Frequency of F4/80^+^ cells isolated from 1.10^6^ PFU ROCV-infected mouse 7 days after infection. (WT 7 days p.i.) Expression of IL-33 on F4/80^+^ gated cells isolated from CNS of mock and 1.10^6^ PFU ROCV-infected mouse, analyzed by flow cytometry—FACS. *Dots* are representative from a single animal (with at least three animals per group). **b**, **c** Expression levels of IL-33 and ST2L (transmembrane form) mRNA in the brain of mice infected with ROCV 1.10^6^ PFU, analyzed by qPCR. Data are representative as the mean of three independent experiments with at least four animals per group. Statistical analysis was performed by unpaired Student *t* test using the software GraphPad Prism

To further characterize the immune response to ROCV infection, we evaluated the expression levels of IL-33 and the levels of mRNA from ST2L (transmembrane form) receptor on CNS cells isolated from ROCV-infected animals. As observed in Fig. 3a, wild-type (WT) BALB/c infected with a lethal dose of ROCV (1.10^6^ PFU) had an increased infiltration of F4/80^+^ cells on CNS at 7 days post-infection. When we analyzed the levels of IL-33 expression by F4/80^+^ cells isolated from the CNS, we found increased IL-33 production following ROCV infection at day 7 (Fig. 3a). Moreover, a kinetic analysis of IL-33 and ST2L receptor mRNA levels on CNS-isolated cells from 1.10^6^ PFU ROCV-infected mice demonstrated a significant IL-33 response at day 5 post-infection and ST2L receptor upregulation at 5 and 7 days following infection (Fig. 3b, c).

### IL-33 deficiency contributes to viral encephalitis aggravation

**Fig. 4:**
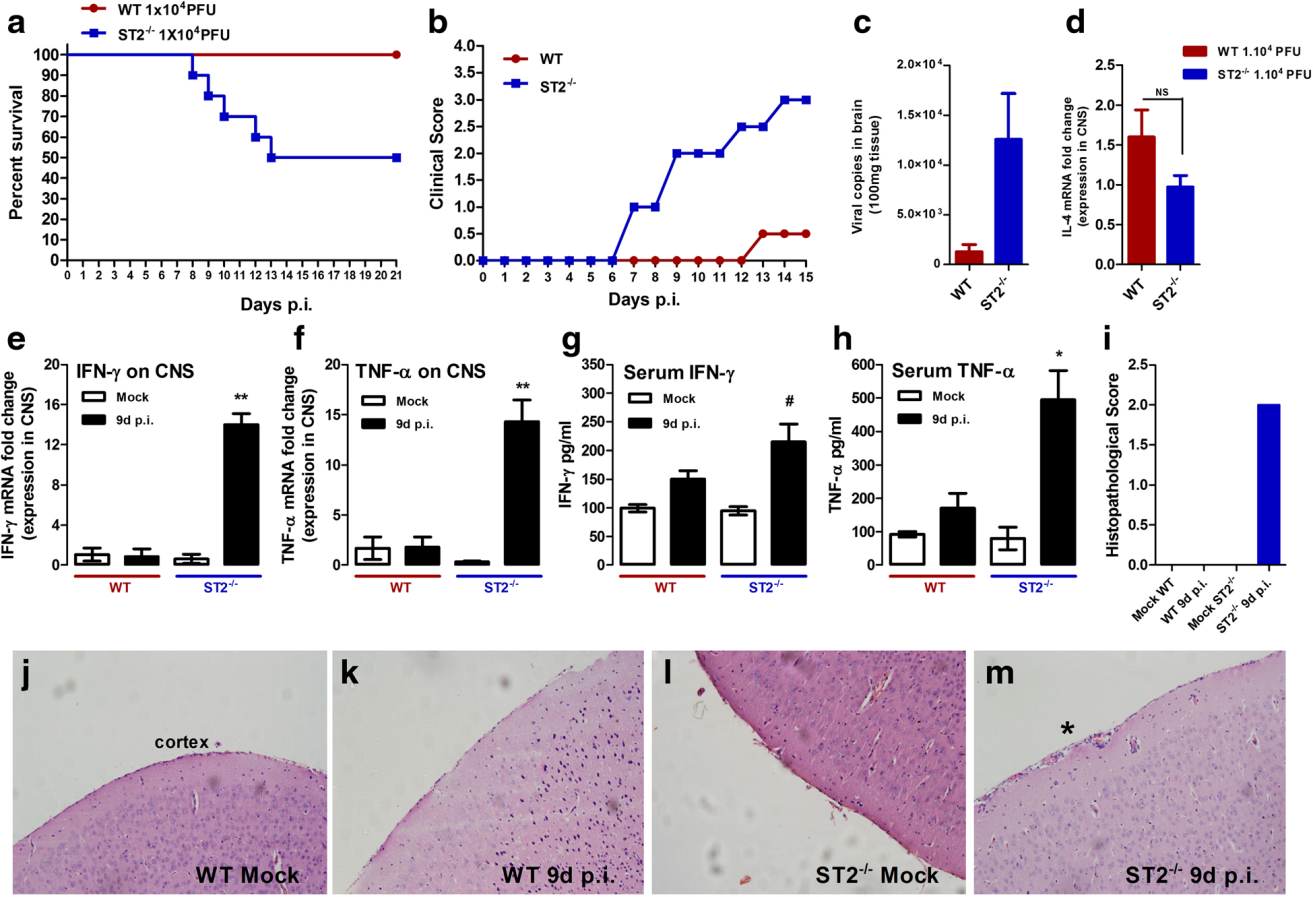
IL-33 protects mice from ROCV-induced encephalitis. **a** Groups of ten female 6-week-old BALB/c (WT) and T1/ST2-deficient mice (ST2^−/−^) were infected intraperitoneally (i.p.) with 1.10^4^ PFU of ROCV and monitored daily for survival up to 21 days post-infection. **b** Clinical score of ROCV-infected WT and ST2^−/−^ mice. **c** The viral load on the brains of infected WT and ST2^−/−^ mice was assessed by real-time qPCR on day 9 post-infection. Expression levels of IL-4 (**d**), IFN-γ mRNA (**e**), and TNF-α (**f**) in the brain of mice infected with ROCV 1.10^4^ PFU 9 days post-infection, analyzed by qPCR. ELISA quantification of IFN-γ (**g**) and TNF-α (**h**) in serum of WT and ST2^−/−^ mice as indicated on the figure legends. **i** Histopathological analysis of brain tissues from mock wild type—histopathological score as described in the “Methods” section. Histologic slides of mock-infected WT (**j**), 1.10^4^ PFU ROCV-infected wild type (**k**), mock ST2^−/−^ (**l**), and 1.10^4^ PFU ROCV-infected ST2^−/−^ mice (**m**); all the animals were sacrificed at 9 days post-infection. Magnification ×200. *p < 0.05, **p < 0.001, or # non-significant compared to WT at 9 days post-infection. Data are representative as the mean of three independent experiments with at least four animals per group. Statistical analysis was performed by unpaired Student *t* test using the software GraphPad Prism

Among several biological activities of IL-33, the promotion of Th2 and inhibition of Th1 responses are well documented [14]. Here, given the strong upregulation of IL-1R4/ST2 observed on CNS from ROCV-infected mice, we aimed to investigate the role of IL-33/ST2 signaling on disease progression. Initially, we infected WT and ST2^−/−^ mice with different concentrations of ROCV, and we observed no differences on survival when the animals were infected with either 1.10^6^ or 1.10^5^ PFU (data not shown). However, when we infected mice by intraperitoneal route with a non-lethal dose of ROCV (1.10^4^ PFU) and compared the disease development on IL-33 receptor-deficient mouse (ST2^−/−^) versus WT mice, we observed that wild-type mice are completely resistant to ROCV-induced encephalitis development; on the other hand, ST2^−/−^ mice experienced a 50 % mortality rate up to 21 days post-infection as observed in Fig. 4a. Also, ST2^−/−^-infected mice presented a higher clinical score beginning as early as 8 days post-infection (Fig. 4b) and a higher viral load on CNS at 9 days post-infection (Fig. 4c) with apparent signs of encephalitis and evident cellular infiltrates at the CNS (Fig. 4g). Next, to elucidate the role of IL-33 on local cytokine modulation, we evaluated the mRNA expression of the Th2 key cytokine IL-4 (Fig. 4d) and pro-inflammatory cytokines IFN-γ (Fig. 4e) and TNF-α (Fig. 4f) in the CNS and by enzyme-linked immunosorbent assay (ELISA) on the serum of infected animals (Fig. 4e, f). As observed, ST2^−/−^ mice had higher levels of TNF-α and IFN-γ transcripts locally (at the CNS) when analyzed 9 days post-infection (when disease symptoms are evident on ST2^−/−^ mice); systemically (serum cytokines), the production of these cytokines were also increased, when compared to WT mice (Fig. 4g, h).

Histopathological analysis of CNS from mock and infected animals demonstrated a higher cellular infiltrate only on ST2^−/−^ animals following 9 days post-infection (Fig. 4i). Inflammatory cell infiltrates were not observed in CNS tissues from uninfected animals and the WT mouse (Fig. 4j–m). The histopathological scores were higher on the ST2^−/−^-infected mouse (Fig. 4h). Mononuclear cellular infiltrates were concentrated mainly on meninges and cortex areas of ST2^−/−^ mice and were more prominent around blood vessels.

### Infiltrating lymphocytes contributes to disease progression by cytokine production

**Fig. 5:**
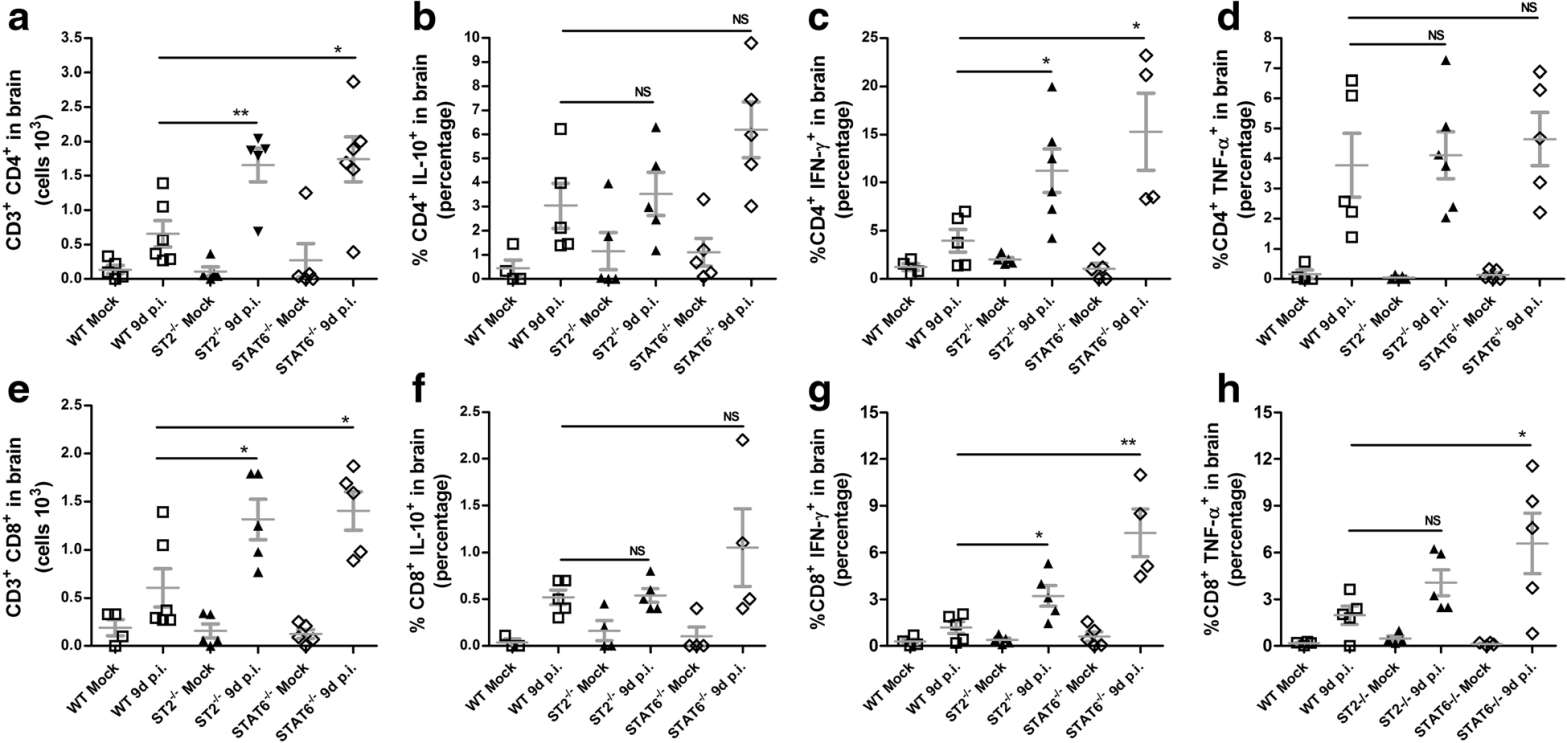
Phenotypic profile of CNS infiltrating cells. BALB/c and ST2^−/−^ mice were infected with 10^4^ PFU of ROCV. CNS infiltrating cells were collected 9 days post-infection, and stimulated for 4 h with phorbol-12-myristate-13-acetate (PMA) and ionomycin in the presence of brefeldin A. The frequency of CD3^+^ and CD4^+^ or CD8^+^ T cells (**a**, **e**) and IL-10 (**b**, **f**), IFN-γ (**c**, **g**), and TNF-α (**d**, **h**)-producing cells was analyzed by flow cytometry (FACS). *p < 0.05, **p < 0.001. *NS* non-significant. Data are representative as the mean of three independent experiments with at least five animals per group. Statistical analysis was performed by Student *t* test using the software GraphPad Prism

To better understand the pathogenic mechanism of IL-33 signaling on ROCV-induced encephalitis, we explored the cellular composition of CNS tissue and the cytokines being produced on WT versus ST2^−/−^ mice. FACS analysis of the CNS tissue 9 days post-infection demonstrated a cellular infiltrate constituted mainly by CD4^+^ and CD8^+^ T cells (Fig. 5a, e). ST2^−/−^-infected mice had an increased frequency of both CD4^+^ and CD8^+^ T cells; moreover, only CD3^+^CD4^+^ cells produce significantly higher levels of IFN-γ (Fig. 5c, g) but not TNF-α (Fig. 5d, h) or IL-10 (Fig. 5d, h) after the infiltrate of the CNS tissue.

### ST2^−/−^ mice show increased iNOS expression in the CNS following ROCV infection

**Fig. 6:**
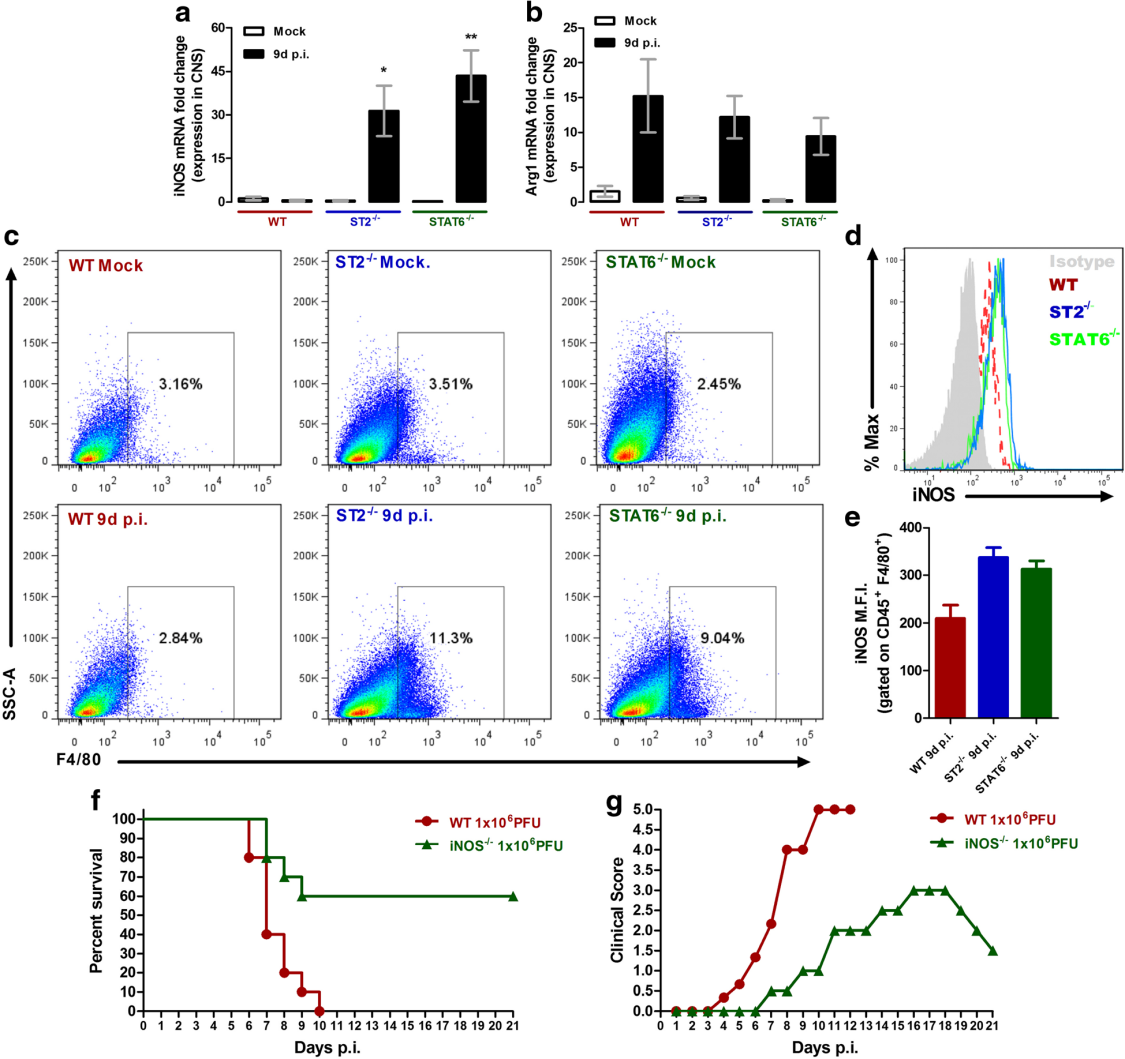
IL-33 and nitric oxide participates on ROCV-induced encephalitis. **a** iNOS mRNA expression in the brain of WT, ST2^−/−^- and STAT6^−/−^-infected mice. **b** Arg1 mRNA expression in the brain of WT,ST2^-/-^ and STAT6-/- infected and control mice. mRNA expression was determined with qPCR normalized for the GAPDH CT value. Data are expressed as mean ± SE. **c** Frequency of F4/80+ cells (gated on CD45+) isolated from brain tissues of WT, ST2^−/−^ and STAT6^−/−^ 9 days after infection with ROCV. **d** iNOS expression (gated on CD45+ F4/80+ cells). **e** Mean fluorescence intensity (MFI) of iNOS+ cells. **f** Groups of ten female 6-week-old WT or iNOS-deficient mice (iNOS^−/−^) were infected intraperitoneally with 1.106 PFU of ROCV and monitored daily for survival up to 21 days post-infection. **g** Clinical score of ROCV-infected mice. Mice were infected with 1.106 PFU of ROCV and sacrificed as indicated. *p < 0.05, **p < 0.001. Statistical analysis was performed by Student t test using the software GraphPad Prism. Data are representative as the mean of three independent experiments with at least four animals per group

In this study, we have found a higher proportion of lymphocytes infiltrating the CNS of ROCV-infected ST2^−/−^ mice at 9 days post-infection, when the disease is evident; additionally, these cells appear to contribute to encephalitis aggravation by producing higher levels of pro-inflammatory cytokines. Thus, we hypothesized that the higher amounts of IFN-γ released on ST2^−/−^ CNS mice could contribute to disease aggravation by shifting the local immune response to a Th1 profile, inducing a debalanced Th1/Th2 immune response and tissue damage. On type 2 innate lymphoid cells (ILC2), IL-33 signaling induces the production of Th2 cytokines such as IL-4, IL-5, and IL-13, leading to M2 macrophage polarization [15]. To check if the absence of IL-33 signaling could be affecting macrophage polarization, we evaluated the expression of the enzyme inducible nitric oxide synthase (iNOS), which metabolizes arginine to nitric oxide (NO) and is highly upregulated on M1 macrophages. Also, given that Stat6 phosphorylation is mediated by IL-4 and that IL-4-mediated inhibition of iNOS expression on macrophages is Stat6 dependent [16], we investigated the potential role of Stat6 in ROCV-induced encephalitis. We analyzed by quantitative polymerase chain reaction (qPCR) the levels of iNOS (M1 macrophage marker) and arginase-1 (M2 macrophages marker) on CNS tissue from infected animals; on both groups (ST2^−/−^- and Stat6^−/−^-infected mice), the mRNA levels for iNOS (Fig. 6a), but not arginase-1 (Fig. 6b), were significantly upregulated at 9 days post-infection, compared to WT infected mice. To check which cells could be contributing to iNOS expression, we performed a FACS analysis of CNS cellular infiltrates. We observed a higher frequency of CD45^+^ F4/80^+^ cells on ST2^−/−^- and Stat6^−/−^-infected mice (1.10^4^ PFU) at 9 days post-infection, when compared to WT mice (Fig. 6c). Moreover, these cells were also expressing much higher levels of iNOS (Fig. 6d, e), analyzed by FACS on CNS-isolated CD45^+^ F4/80^+^ gated cells. To complement our data, we checked survival and clinical score of Stat6^−/−^ mice and we observed similar results to ST2^−/−^-infected mice, as well CNS-infiltrating lymphocyte profile and serum cytokines (Additional file 1: Figures S1 to S4).

### iNOS deficiency protects mice from lethal viral encephalitis

Finally, to check the role of NO on ROCV-induced experimental encephalitis, we infected iNOS^−/−^ and WT C57BL/6 mice with a lethal dose of ROCV (1.10^6^ PFU) and monitored survival, clinical score, and tissue pathology on these animals. As observed in Fig. 6f, iNOS^−/−^ mice presented an increased survival rate than wild-type infected animals. Moreover, iNOS^−/−^ mice were partially protected from disease as evidenced by the lower clinical score (Fig. 6g). Interestingly, iNOS^−/−^ mice CNS exhibited abundant cell infiltrates to levels compared to WT mice (Additional file 1: Figure S5) despite the increased survival rate.

## Discussion

Understanding the pathogenic mechanisms of viral infections in the CNS is critical to encephalitis treatment. Previous reports demonstrate that CNS IL-33 signaling is related to protection following experimental infection with *Toxoplasma gondii*, and the authors found that IL-33 deficiency leads to increased parasite burden and pathology in the brains of infected mice [17]. Here, in this study, we demonstrate that IL-33 has a protective role on experimental viral encephalitis. Our data supports previous studies in the literature demonstrating the contribution of IL-33 on T cell response modulation [7, 18]. Here, the absence of ST2 (the receptor for IL-33) following the infection with an encephalitogenic virus leads to increased cellular-mediated immunopathology on mouse CNS.

Although the influenza A virus induced the production of IL-33 in alveolar macrophages and the activation of IL-13-producing natural helper cells and that after infection with coxsackievirus B, IL-33 in the pancreas is markedly elevated, data about the role of IL-33 signaling on viral infections are still scarce [8, 19]. Even though the participation of IL-33 is well documented on several different pathologies and disease conditions, we are the first to demonstrate the protective role of IL-33 signaling on viral experimental encephalitis. We are showing that IL-33 is expressed on the CNS following infection with a neurotropic virus, balancing the local immune response with consequent protection from exacerbated CNS injury. In fact, among other CNS pathologies, Jiang et al. [20] demonstrated that IL-33 administered after experimental autoimmune encephalomyelitis (EAE) onset could be protective against the disease and also that ST2^−/−^ mice developed exacerbated disease following EAE induction. Interestingly, recombinant IL-33 treatment attenuates EAE by suppression of IL-17 and interferon-gamma (IFN-γ)-producing cells [20]. In addition, it was recently demonstrated that IL-33 is widely expressed on the healthy brain, especially by oligodendrocytes and gray matter astrocytes. Following CNS injury, induced by mechanical spinal cord contusion, IL-33 is immediately released acting on astrocytes and microglia to signal the production of chemokines that recruit monocytes and enhance M2 genes at the injury site involved in immunosuppression and tissue repair [21]. Thus, it seems that IL-33 production in response to CNS injury could represent a protective mechanism. In our model, the protective role of IL-33 is directly related to the local T cell response modulation, since IL-33 deficiency leads to augmented Th1 local immune response, with IFN-γ overproduction and increased death following infection with ROCV. In fact, on viral infections, IFN-γ is important to virus clearance; however, exacerbated IFN-γ response could lead to tissue damage due to the extensive inflammation process, as observed in our results.

Given that IL-33 is characterized as a nuclear alarmin released after the cell is damaged, it is not surprising that a neurotropic virus such as ROCV could upregulate IL-33 production. ROCV infection induces degeneration and neuronal death, and apoptotic cells expressing caspase 3 (mainly neurons, lymphomononuclear and endothelial cells) were significantly increased across different CNS regions in later time points after ROCV infection. Viral antigens are usually detected on CNS after 4 days of infection [2]. In our results, we detected IL-33 expression also in later time points, coinciding with the detection of virus replication in the CNS. However, virus replication itself may not be the only factor inducing cell damage in the CNS; we suggest that the extensive injury might actually be a consequence of the inflammation, characterized by waives of cellular infiltration and local cytokine production. In this scenario, IL-33 is important to drive the expression of Th2-type cytokines which in turn can protect the animals by downregulating the CNS Th2-type inflammatory response. On the other hand, Th2 cytokines might not have a direct role on viral clearance. In fact, on viral infections of the CNS, the immune responses implicated on viral clearance are usually cell specific, for example, antibody responses are important to neurons and the T cell-mediated immune responses are more important to virus clearance from glial cells [22]. Upon ROCV infection, a mixed Th1/Th2 immune response is already documented [2]. Thus, it is crucial to identify specific immune factors that participate in protective virus response. In our analyses, we demonstrate a critical protective role of IL-33 signaling on CNS immune responses. However, the precise mechanisms of IL-33 synthesis, localization, and release during CNS viral inflammation remain to be fully characterized. Although we indentify macrophages expressing higher levels of IL-33 after infection, we cannot exclude that other CNS resident cells such as glial cells and astrocytes could be the main source of IL-33 production. In fact, it is previously demonstrated that astrocytes secrete IL-33 after inflammatory stimulation with TNF-α in vitro and that damaged neurons actively secrete IL-33 following EAE [23]. Moreover, it was recently demonstrated that dendritic cells can produce IL-33 after engagement of TLR4 and TLR5 receptors, and this leads to NF-kB activation and IL-33 expression following microbial stimulus [24]. The authors testify that the dendritic cell IL-33 could amplify local inflammatory response through an autocrine mechanism. More recently, Kim et al. [25] demonstrated that CD11c^hi^ dendritic cells act on CNS by controlling the activation and function of infiltrated monocytes. By selective deletion of CD11c^hi^ dendritic cells, the authors found increased viral copies on CNS and death following mice infection with Japanese encephalitis virus; further analysis leads to the conclusion that CD11c^hi^-DC cells can regulate the activation of CD11b^+^Ly-6C^hi^ monocytes controlling exacerbated CNS inflammation [25]. In our study, the participation of dendritic cells was not addressed and further experiments are necessary to investigate the role of these cells on viral encephalitis.

Several different neurotropic viruses such as rabies virus (RABV), Japanese encephalitis virus (JEV), and West Nile virus (WNV) and more recently Zika virus present the potential to disrupt the blood brain barrier (BBB) and establish tissue damage [26–28]. The pathogenic mechanisms that contribute to BBB disruption are diverse, and chemokines or inflammatory cytokines such as IFN-γ, IL-8, TNF-α, and IL-1β can indirectly contribute [29]. In this context, IFN-γ has an important role. Following infection with JEV on the mouse, neutralization of IFN-γ ameliorated the enhancement of BBB permeability. However, the administration of IFN-γ-neutralizing antibody did not significantly change the viral load on CNS [27]. Thus, it seems that IFN-γ production on CNS could act by breaking the BBB allowing or facilitating the infiltration and activation of resident cells such as microglia. Interestingly, microglia activation has been suggested to be responsible for the progression of neuronal damage-releasing neurotoxic factors such as TNF-α or nitric oxide (NO) [30]. In conclusion, given that all these pro-inflammatory factors are cytotoxic to neurons, IL-33 signaling on CNS is crucial to balance the local inflammatory process by avoiding excessive pro-inflammatory cytokine production, protecting the neurons from extensive damage. In our model, we observed higher levels of both mediators: IFN-γ and NO, following infection of IL-33 signaling-deficient mouse (ST2^−/−^) with ROCV. Thus, microglia overactivation through NO production could serve to enhance or amplify the neuronal damage induced by virus replication. Additionally, we observed a higher viral load on the CNS of ST2^−/−^ mice; this could be partly explained by the fact that the IL-33-deficient mouse presented higher frequencies of cellular infiltrates in the brain (providing more cells that can be infected). On the other hand, IL-33 signaling is necessary to generate effective CD8^+^ T cell responses in viral and tumor metastatic models [12, 31]. Thus, ineffective cytotoxic T cell lymphocyte (CTL) response (CTL) by the lack of IL-33 could favor ROCV replication in our model, although a more detailed study on CTL function could provide more solid data.

Numerous pathological conditions such as Alzheimer’s disease, Parkinson’s disease, and multiple sclerosis are well known to induce neurodegeneration [32]. The mechanisms of neuroinflammation are dependent on glial cell activation, which releases a number of neurotoxic factors, especially nitric oxide species (NOS) and cytokines. In the CNS, the production of pro-inflammatory molecules results in apoptosis in various subsets of resident brain cells. Interestingly, NO has a neuroprotective effect at physiological levels; however, NO becomes harmful if it is produced in excess, leading to neuronal damage [33]. Usually, iNOS expression in the CNS is very low and can be induced in astrocytes or microglial cells following events such as viral infection. In fact, it has been reported that in vitro infection of mouse macrophages with ROCV leads to NO production [34]. Here, the induction of NO release by virus infection is highly amplified on the ST2^−/−^ mouse as a direct consequence of augmented Th1 response on the CNS. This was associated with the increased CNS infiltration of immune cells, particularly Th1 cells. Higher levels of IFN-γ can act by shifting infiltrating macrophages to the M1 phenotype, as evidenced by a higher expression of iNOS transcripts on infected mouse CNS. M1 macrophages are cytotoxic and exhibit pro-inflammatory markers, characterized by the production of pro-inflammatory cytokines and high levels of reactive oxygen and nitrogen species [35]. Importantly, it was recently reported that the lack of IL-33 signaling in CNS results in lower levels of infiltrating neuroprotective M2 macrophages, which, in turn, leads to impaired recovery after CNS mechanical injury [21]. Thus, here we provide evidence that following viral infection, IL-33 is produced in the CNS to counteract the deleterious effects of pro-inflammatory cytokines IFN-γ and TNF-α, as well to shift macrophage response to a M2-protective phenotype.

We previously demonstrated that ROCV infection results in increasing diffuse neuronal degeneration starting at 4 days post-infection, with positive caspase 3 glial and neuronal cells at 9 days post-infection [2]. Nitric oxide production in the CNS is highly toxic to neurons. Thus, we hypothesized that NO release in the CNS could represent the most downstream event on IL-33 local response modulation. Another function of NO is to act as a negative regulator of immune cell trafficking to the CNS [36]. It is very interesting that the macrophage from the M1 phenotype infiltrates injured CNS tissue via CCL2 signaling through adjacent spinal cord leptomeninges; on the other hand, M2 cells came from monocytes that trafficked through the brain-ventricular choroid plexus (CP), via the VCAM-1-VLA4 and CD73 mechanism [37]. At the CP, NO inhibits NF-kB/p65 translocation to the nucleus and sequential leukocyte entry to the CNS [36]. In our results, we observed higher cellular infiltrates in iNOS^−/−^-infected mice (Additional file 1: Figure S5); however, iNOS deficiency results in increased survival rates following ROCV infection despite the higher pathological scores. Thus, taken together, our data support the idea that NO production in the CNS is the most neurotoxic product.

## Conclusions

Taken together, our data indicate a direct role of IL-33 signaling on CNS immunopathology control. The absence of IL-33 signaling increases the CNS inflammation by a mechanism dependent on IFN-γ production that leads to NO production and consequent tissue damage. Finally, the data presented here may contribute to the design of new therapies to viral encephalitis based on the benefits of IL-33 administration or the blockade of its receptor ST2 to avoid extensive damage elicited by the immune response to CNS insults.

## Methods

### Virus

ROCV (SPH34675 strain) was used for mouse infection and kindly provided by Prof. Dr. Luiz Tadeu M. Figueiredo, Ribeirão Preto School of Medicine, University of São Paulo—Brazil. Virus stocks were obtained from the brains of intracerebrally infected suckling mice. Mouse brains were aspirated and macerated in phosphate-buffered saline (PBS) and centrifuged at 500*g* for 10 min at 4 °C, and supernatants were collected and stored at −70 °C until use. Viral stocks were titrated by the plaque-forming unit (PFU) assay, as previously described [38].

### Animal experimentation

Wild-type BALB/c (WT), ST2-deficient (T1/ST2^−/−^), and Stat6-deficient (Stat6^−/−^) mice were infected by intraperitoneal route with different concentrations of ROCV stocks, as stated. C57/B6 wild type and iNOS^−/−^ were infected by intraperitoneal route of ROCV. A mock control group (false infected) was inoculated with extracts from uninfected brain in PBS/albumin7.5 % by the same route, and volume as used for the virus infection The clinical score was monitored daily, and the animals were scored accordingly to the following: 0 normal mouse, no overt signs of disease; 1 limp tail or hind limb weakness; 2 limp tail and hind limb weakness; 3 partial hind limb paralysis; 4 complete hind limb paralysis; and 5 moribund state, death by encephalitis (sacrifice for ethical reasons). Survival was monitored daily up to 21 days. For tissue collection, the animals were sacrificed on different days post-infection (p.i.) as indicated and perfused and tissues samples were collected for analysis. A blood sample was collected to quantify the levels of serum cytokines by ELISA. The animals were housed at five per cage with food and water available ad libitum. All experiments were conducted in accordance with the prescribed guidelines on experimental animal welfare of the National Institutes of Health and were approved by the Ethics Committee of the Ribeirão Preto Medical School, University of São Paulo.

### Histopathological analysis and CNS histologic scores

Histologic scores were performed in the CNS of infected and control mice at 9 days post-infection. Briefly, animals were euthanized and perfused, and brain tissue was removed and fixed in 10 % formaldehyde. Tissues were dehydrated in graded ethanol and embedded in a 100 % paraffin block. Serial sections with 5-μm thickness were prepared and stained with hematoxylin and eosin. Five to six photos were obtained for each animal. The extent of meningeal inflammation was assessed and graded as follows: 0, no inflammation (total absence of inflammatory cells at the meninges area); 1, one cell layer of inflammation (a single layer of infiltrating cells at the meninges); 2, two cell layers of inflammation (two cell layers at the meninges area); 3, three cell layers of inflammation; 4, four cell layers; and 5, five or more cell layers of inflammation. The area with the maximal extent of tissue damage was used for the assessment of each brain region.

### Clinical scores

The clinical score was assessed daily as described by Miller et al. [39]. Briefly, the following grades were considered: 0 normal mouse, no signs of disease; 1 limp tail or hind limb weakness (but not both); 2 limp tail and hind limb weakness (both symptoms manifestation); 3 partial hind limb paralysis; 4 complete hind limb paralysis; and 5 moribund state (euthanasia performed for ethical issues).

### Tissue quantification of cytokine mRNAs and viral load

Total RNA from brain samples (approximately 100 mg from each animal time point) was extracted with the TRIzol Reagent (Life Technologies, Carlsbad, CA, USA) according to the manufacturer’s instructions. After extraction, RNA was quantified by absorbance at 260 nm and approximately 1 μg of each sample was used to obtain the complementary DNA (cDNA) strands using the SuperScript III enzyme (Life Technologies) and Oligo(dT)12-18 for first strand synthesis, according to the manufacturer’s instruction. Real-time polymerase chain reaction (PCR) analysis of cytokine mRNA profiles was performed with the Power SYBR® Green Master Mix kit (Applied Biosystems, CA, USA), and reactions were processed in ABI7500 equipment (Applied Biosystems). The results were analyzed on the basis of the cycle threshold (Ct) of target genes, and the level of expression was calculated by comparing to an endogenous control gene (β-actin) using the ΔΔCt method (relative amount of target gene for each sample is quantified by the equation 2^−ΔΔCt^); data were analyzed with the comparative Ct method. The reactions were carried out in a final volume of 25 μl containing 12.5 μl of SYBR Green PCR Master Mix, 0.5 μM of each primer (designed on the basis of the mRNA sequence of the cytokine genes), and 5 μl of cDNA. RT-PCR cycles were 95 °C for 15 s and 60 °C for 1 min, and the dissociation curve was constructed by increasing temperatures from 60 to 90 °C. Viral copies were determined by qPCR, as described above. A standard curve from a previous established virus stock was serially diluted to construct a standard curve, and viral copies were determined from each sample analyzed; results were expressed as viral copies in the brain per 100 mg/tissue. Primers used for cytokine quantification are listed in Table 1.

**Table 1:** Primers used for gene expression analysis

Targets	Primer sequences (5′ ≥ 3′)
IFN-γ	F: ATCAGGAGGGACTCCTTTTCCGCTT
	R: GAAGCCTAGAAAGTCTGAATAACT
IL-17A	F: GGTCAACCTCAAGTCTTTAACTC
	R: TTAAAAATGCAAGTAAGTTTGCTG
TNF-α	F: ACCAGCTAAGAGGGAGAGAAGCAA
	R: TCAGTGCTCATGGTGTCCTTTCCA
IL-33	F: TCCTTGCTTGGCAGTATCCA
	R: TGCTCAATGTGTCAACAGACG
ST2L	F: CATGGCATGATAAGGCACAC
	R: GTAGAGCTTGCCATCGTTCC
iNOS	F: CAGTTCCGAGCGTCAAAGACCTGC
	R: CAGCCCAACAATACAATACAAGATG
arg1	F: GTTCCCAGATGTACCAGGATTC
	R: CGATGTCTTTGGCAGATATGC
IL-4	F: GAATGTACCAGGAGCCATATC
	R: CTCAGTACTACGAGTAATCCA
β-actin	F: AGCTGCGTTTTACACCCTTT
	R: AAGCCATGCCAATGTTGTCT
ROCV—SPH34675—NS5	F: GGTCAATGCCACAAGCCAAG
	R: CTTCAGCCTTTCGATCCGGT

### Viral titration

Viral stocks were titrated by the PFU assay. Briefly, Vero cells were cultivated in 24-well plates, and cell-confluent monolayers were infected with 100 μl of serial dilutions of the viral stocks. Next, the cells were incubated for 1.5 h at 37 °C and washed with PBS, and an overlay consisting of DMEM containing 2 % fetal bovine serum and 3 % carboxymethylcellulose was added to the wells. The cells were then incubated for 7 days at 37 °C/5 %CO_2_. After this period of incubation, the overlay was discarded and the cells were washed with PBS and stained with 0.5 % Neutral Red solution. The viral titer was based on the number of plaques present in the highest dilution.

### Cytokine quantification ELISA

Serum samples were used to quantify the levels of IFN-γ and TNF-α using commercial kits (R&D Systems, Abingdon, UK). ELISA was performed by coating 96-well polystyrene microtiter plates with the specific antibody. After blocking, standards and samples were and the plates were incubated overnight at 4 °C. A specific biotinylated antibody was added to all wells and incubated for 1 h at room temperature. The plates were washed and incubated for 30 min with horseradish peroxidase-conjugated streptavidin. 3-3′,5,5′-tetramethylbenzidine (TMB) substrate reagent solution (Sigma-Aldrich) was added to the wells. Absorbance was read at 450 nm.

### Flow cytometry

Mice were perfused with PBS before the brains were harvested. Brain tissues were pretreated with 2 μg/ml collagenase D and 1 μg/ml DNAse I (both Roche Diagnostics), and total cells were isolated by cell straining (100-μm mesh). Brain homogenates were separated into neuronal and leukocyte populations by discontinuous density gradient centrifugation using isotonic Percoll (GE HealthCare, Uppsala, Sweden) as described by Pino and Cardona [40]. After isolation, cells were stimulated in vitro with PMA and Ionomycin plus BD GolgiStop™ (BD Biosciences, San Jose, CA, USA) for at least 4 h at 37 °C. After stimulation, the cells were fixed with BD Cytofix/Cytoperm™ Plus Fixation/Permeabilization Kit and intracellular staining was carried out following the manufacturer’s instructions (BD Biosciences). Flow cytometry was performed using a FACS Verse (BD Biosciences) with the specific antibodies listed: anti-CD45 clone A20, anti-CD4 clone GK1.5, CD8α clone 5H10-1, IFN-γ clone XMG1.2, TNF-α clone MP6-XT22, CD3 clone 17A2, IL-10 clone JES5-16E3, F4/80 clone BM8 (all from BioLegend Inc., San Diego, CA, USA), IL-33 clone 396118 (R&D Systems, Minneapolis, MN, USA), and anti-iNOS (NOS2) clone N-20 (Santa Cruz Biotechnology, San Diego, CA, USA).

### Statistical analysis

The data are reported as the means ± SEM. Student’s *t* test was used to test for statistical significance of the differences between the different group parameters. *p* values of less than 0.05 were considered statistically significant. When necessary, statistical analysis was performed by either one-way ANOVA or two-way ANOVA with Bonferroni post-test as stated on figure legends.

## Abbreviations

BBB, blood brain barrier; CCL2, chemokine (C-C motif) ligand 2; CCR5 C-C, chemokine receptor type 5; CD11b, cluster of differentiation 11b; CD11c, cluster of differentiation 11c; CD3, cluster of differentiation 3; CD4+, cluster of differentiation 4; CD45+ cluster of differentiation 45; CD73, cluster of differentiation 73; CD8+, cluster of differentiation 8; cDNA, complementary DNA; CNS, central nervous system; CP, choroid plexus; CTL, cytotoxic T lymphocyte; DMEM, Dulbecco’s minimal essential media; EAE, experimental autoimmune encephalomyelitis; ELISA, enzyme-linked immunosorbent assay; FACS, fluorescent-activated cell sorting; IFN-γ, interferon-gamma; IL-10, interleukin 10; IL-13, interleukin 13; IL-17A, interleukin 17A; IL-1β, interleukin 1 beta; IL-33, interleukin 33; IL-4, interleukin 4; IL-5, interleukin 5; IL-8, interleukin 8; iNOS, inducible nitric oxide synthase; JEV, Japanese encephalitis virus; Ly-6C, lymphocyte antigen 6 complex; MIP-1α, macrophage inflammatory protein 1 alpha; mRNA, messenger ribonucleic acid; MyD88, myeloid differentiation primary response gene 88; NF-kB, nuclear factor kappa b; NO, nitric oxide; p.i., post-infection; PBS, phosphate-buffered saline; PCR, polymerase chain reaction; PFU, plaque forming unit; qPCR, quantitative polymerase chain reaction; RABV, rabies virus; RNA, ribonucleic acid; ROCV, Rocio virus; RT-PCR, reverse-transcription polymerase chain reaction; Th1, T helper 1; Th2, T helper 2; TNF-α, tumor necrosis factor alpha; VCAM-1, vascular cell adhesion protein 1; VLA-4, very late antigen 4; WNV, West Nile virus; WT, wild type.

## Additional file

Additional file 1: Figure S1. Phenotypic profile of CNS-infiltrating cells on Stat6^−/−^ mice. **Figure S2**. Stat6^−/−^ mice survival after ROCV infection. **Figure S3**. Cytokine profile on Stat6^−/−^ mice. **Figure S4**. Effect of ROCV infection on transaminases and urea profile on WT, ST2^−/−^, and Stat6^−/−^ mice. **Figure S5**. Nitric oxide deficiency leads to increased histopathological score on CNS ROCV-induced encephalitis. **Figure S6**. Gating strategy for macrophage F4/80+ cell analysis.
