# Supplementary material for: IL-33 signaling is essential to attenuate viral-induced encephalitis development by downregulating iNOS expression in the central nervous system
Source: J Neuroinflammation. 2016 Jun 22;13:159. doi: 10.1186/s12974-016-0628-1 (PMC4917985; doi:10.1186/s12974-016-0628-1)
Supplement: Additional file 1: Figure S1. — Phenotypic profile of CNS-infiltrating cells on Stat6−/− mice. Figure S2. Stat6−/− mice survival after ROCV infection. Figure S3. Cytokine profile on Stat6−/− mice. Figure S4. Effect of ROCV infection on transaminases and urea profile on WT, ST2−/−, and Stat6−/− mice. Figure S5. Nitric oxide deficiency leads to increased histopathological score on CNS ROCV-induced encephalitis. Figure S6. Gating strategy for macrophage F4/80+ cell analysis. [file 12974_2016_628_MOESM1_ESM.docx]

**SUPLEMMENTARY DATA**

**IL-33 signalling is essential to attenuate viral induced encephalitis development by downregulating iNOS expression in the central nervous system**

Rafael F.O. Franca^1#^**^*^**, Renata S. Costa^2^**^*^**, Jaqueline R. Silva^2^, Raphael S. Peres^2^, Leila R. Mendonça^1^, David F. Colón^2^, José Carlos Alves-Filho^3^ and Fernando Q. Cunha^3#^


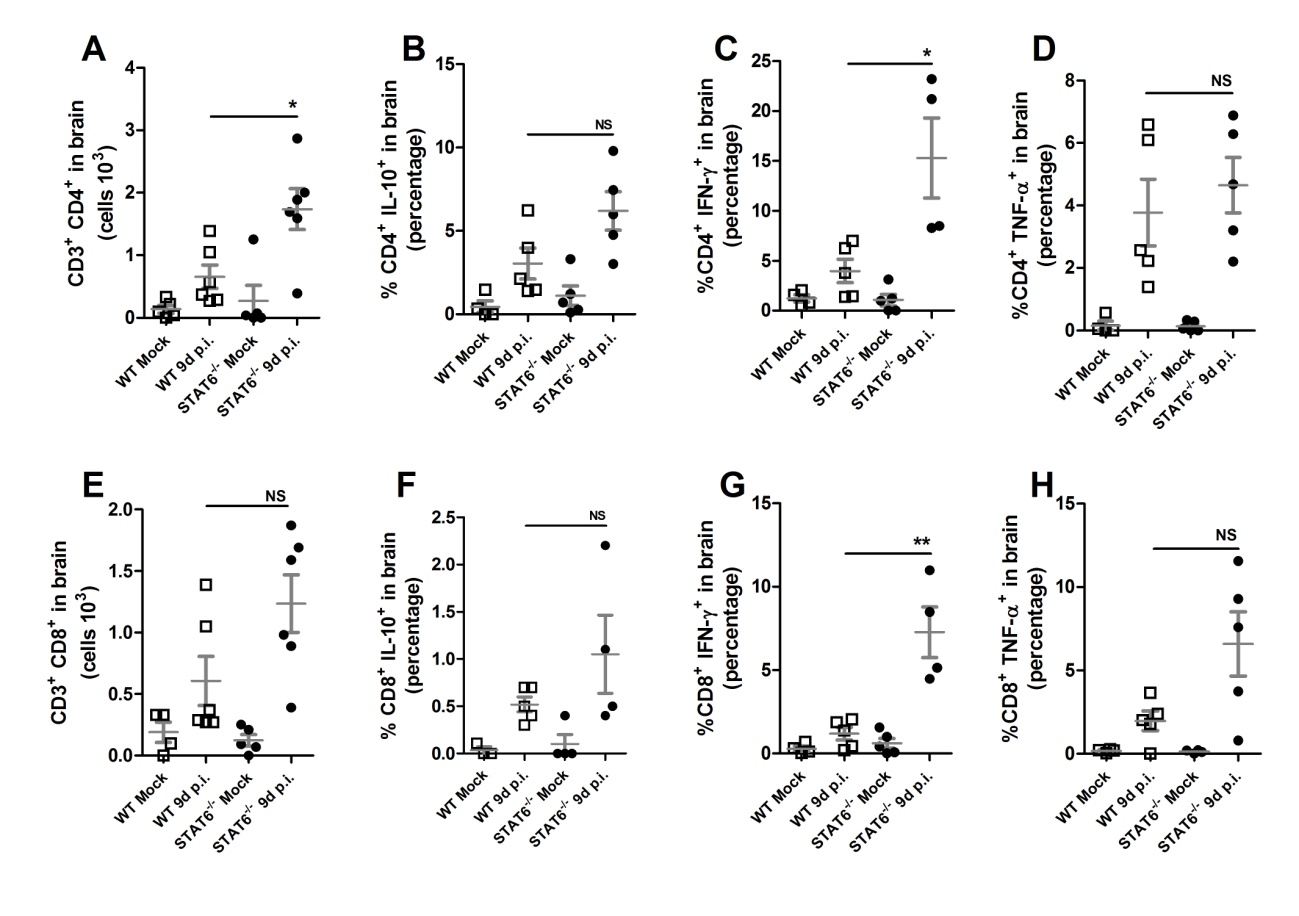


**Figure S1. Phenotypic profile of CNS infiltrating cells on Stat6^-/-^ mice.** BALB/c and Stat6^-/-^ mice were infected with 10^4^ PFU of ROCV. CNS infiltrating cells were collected 9 days post infection, and stimulated with phorbol-12-myristate-13-acetate (PMA) and ionomycin in the presence of brefeldin A. The frequency of CD3^+^ and CD4^+^ or CD8^+^ T cells (A and E) and IL-10 (B and F), IFN-γ (C and G) and TNF-α (D and H) producing cells was analyzed by flow cytometry (FACS). *p<0.05, **p<0.001. NS non-significant. Data are representative as the mean of three independent experiments with at least five animals per group. Statistical analysis was performed by Student t-test using the software GraphPad Prism.


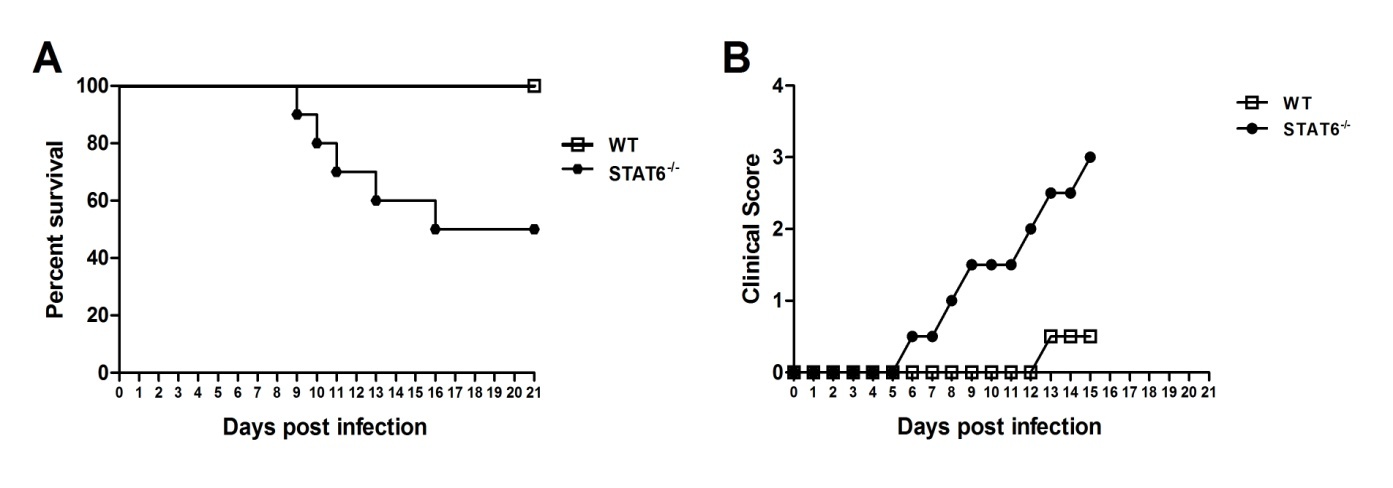


**Figure S2. Stat6^-/-^ mice survival after ROCV virus infection**. (A) Groups of ten female 6-week-old Bab/C (WT) and Stat6^-/-^ mice were infected intraperitoneally (i.p.) with either 1.10^4^PFU of ROCV and monitored daily for survival up to 21 days post-infection. (B) Infected mice were monitored daily for weight loss after infection. Results are representative of ten animals per group. Statistical analysis was performed by Student t-test using the software GraphPad Prism.


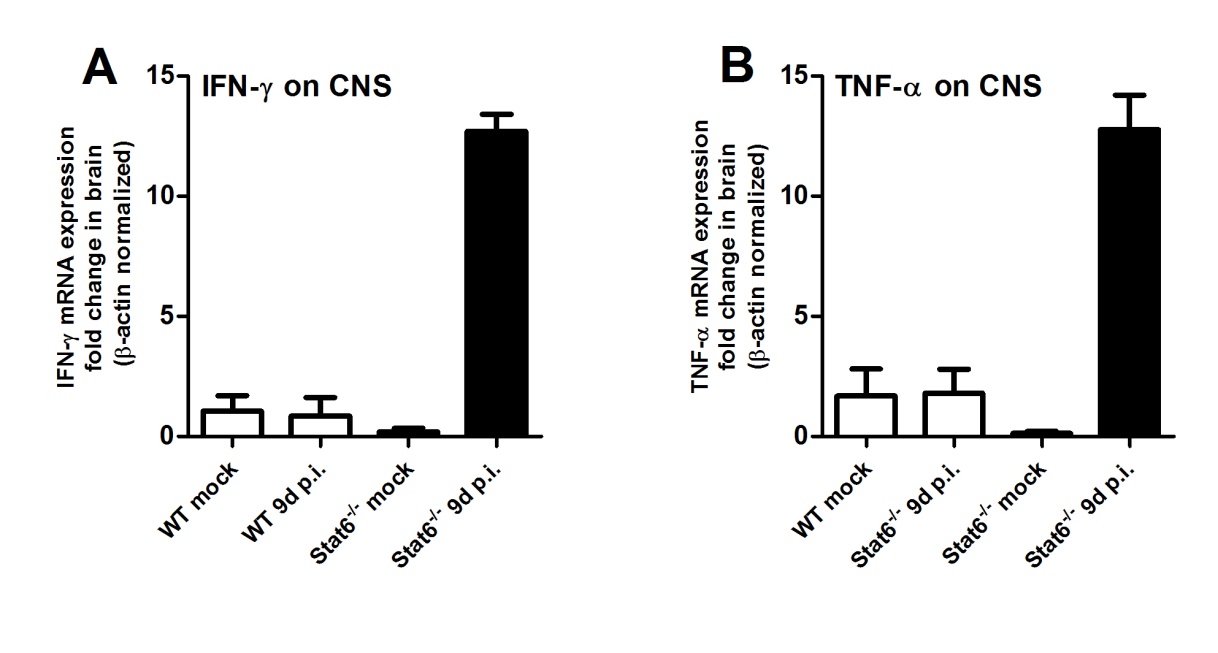


**Figure S3. Cytokines profile on Stat6^-/-^ mice.** (A) Groups of ten female 6-week-old Bab/C (WT) and Stat6 deficient mice (Stat6^-/-^) were infected intraperitoneally (i.p.) with 1.10^4^PFU of ROCV and sacrificed at 9 days post-infection. (A) Expression levels of IFN-γ mRNA, assessed by qPCRand TNF-α (B) in brain of mice infected with ROCV as indicated. Data are representative as the mean of three independent experiments with at least four animals per group. Statistical analysis was performed by Unpaired Student t-test using the software GraphPad Prism.


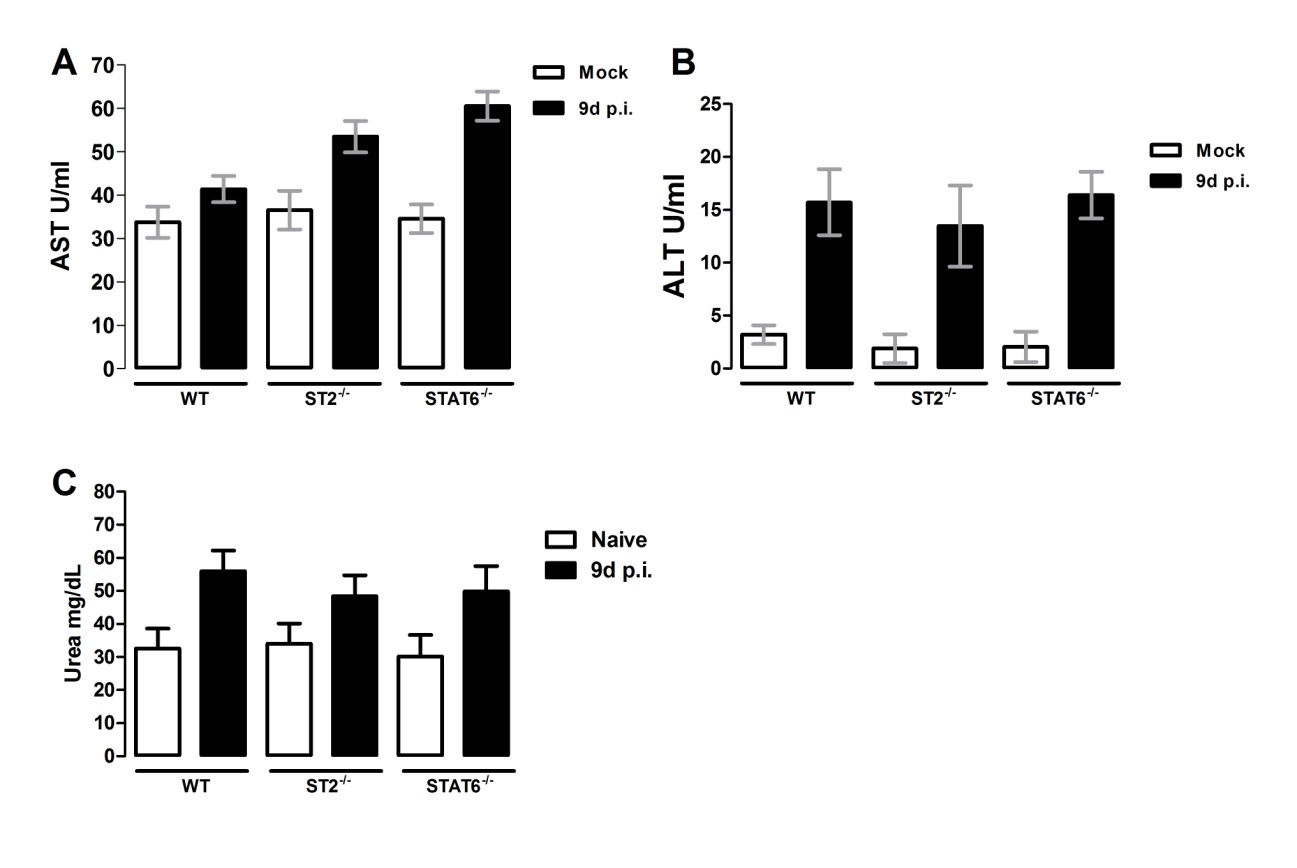


**Figure S4. Effect of ROCV infection on transaminases and urea profile on WT, ST2^-/-^ and Stat6^-/-^ mice.** Groups of ten female 6-week-old Bab/C WT, ST2 and Stat6 deficient mice were infected intraperitoneally (i.p.) with 1.10^4^PFU of ROCV and sacrificed at 9 days post-infection. (A) Production levels of ALT (aspartate aminotransferase) (A) and AST (serum alanine aminotransferase) (B) on serum from non-infected and ROCV 1.10^4^PFU infected mouse The levels of ALT (alanine aminotransferase) and AST (aspartate aminotransferase) were determined in serum samples from the animals using a commercial kit (Labtest Diagnostica, Vista Alegre, MG - Brazil). The assays were carried out using 10μl of serum from each animal according to manufacturer instructions, and the readings were processed in a spectrophotometer at 510 nm. (C) Urea levels of on serum from non-infected and ROCV 1.10^4^PFU infected mouse. Urea levels were determined on serum from infected and control groups as described, the commercial kit Ureia from LAbtest Diagnostica, Vista Alegre, MG – Brazil was employed on this assay. Data are representative of least five animals per group. Statistical analysis was performed by Unpaired Student t-test using the software GraphPad Prism.


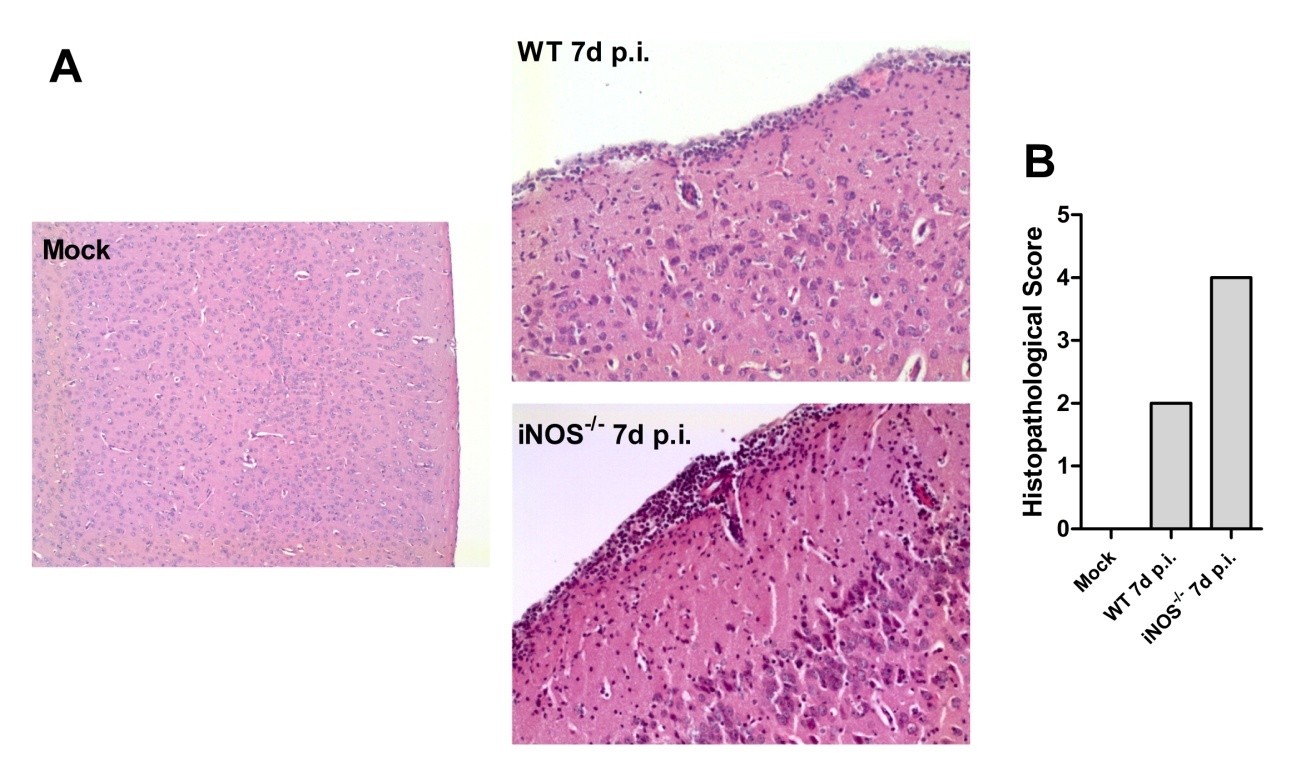


**Figure S5. Nitric oxide deficiency leads to increased histopathological score on CNS ROCV induced encephalitis.** (A) Histopathological analysis of brain from mock infected WT mice (absence of cellular infiltration), WT and iNOS^-/-^ 7 days post infection. Mice were infected with 1.10^6^PFU of ROCV and sacrificed as indicated. (B) Histopathological score as described on material and methods. Magnification 200x.

**
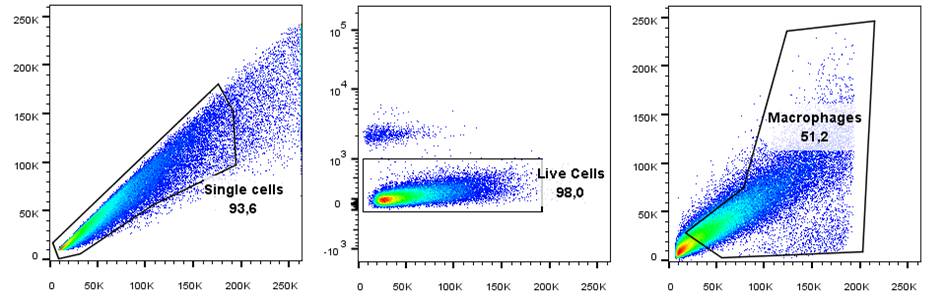
**

**A**

**Figure S6. Gating strategy for Macrophage F4/80+ cells analysis.** (A) Gating strategy to detect F4/80+ IL-33 producing cells isolated from the central nervous system from infected and control groups mice; one representative plot is shown.
